# The role of the ubiquitin-editing enzyme A20 in diseases of the central nervous system and other pathological processes

**DOI:** 10.3389/fnmol.2015.00021

**Published:** 2015-06-15

**Authors:** Asghar Abbasi, Kirsi Forsberg, Felix Bischof

**Affiliations:** Department of Neuroimmunology, Hertie Institute for Clinical Brain Research and Center of Neurology, University Hospital TübingenTübingen, Germany

**Keywords:** A20, NF-κB, neuroinflammation, central nervous system, neurodegenerative diseases

## Abstract

In recent years, the ubiquitin-editing enzyme A20 has been shown to control a large set of molecular pathways involved in the regulation of protective as well as self-directed immune responses. Here, we assess the current and putative roles of A20 in inflammatory, vascular and degenerative diseases of the central nervous system and explore future directions of research.

## General Introduction

The ubiquitin-editing enzyme A20 has now emerged as a key negative regulator of the family of nuclear factor-κB) transcription factors, which control a broad range of cellular activities including cell activation, development and differentiation. NF-κB transcription factors play a central role in the regulation of protective as well as self-directed immune responses and the pathogenesis of human diseases including neurodegenerative diseases and aging. This review focuses on the role of A20 in cellular hemostasis and CNS diseases. NF-κB transcription factors are dimeric proteins composed of the Rel proteins RelA, c-Rel, RelB, p100/p52 and p105/p50. They can be activated via canonical and a non-canonical pathways. In lymphocytes, canonical NF-κB signaling is initiated by antigen-specific B and T cell receptors, Toll-like receptors and other pattern recognition receptors and induces rapid and transient effects such as cell activation and cell-type specific effector functions. The hallmark of the canonical pathway is the signal-induced proteasomal degradation of the mainly cytoplasmic NF-kB inhibitor IκBα. In contrast, non-canonical NF-κB signaling is mediated through specific members of the TNFR superfamily such as CD40, B cell-activating factor belonging to the TNF family receptor (BAFFR), lymphotoxin β-receptor and receptor activator for NF-κB (RANK), which induce NF-κB activation to regulate more persistent cellular functions such as the formation of lymphoid organs, cell survival and cell differentiation. The non-canonical pathway is characterized by the signal-induced processing of the p50-precursor p100, which also has inhibitory functions. RelB-containing NF-kB dimers are tightly regulated by a distinct subset of genes. The activation of NF-κB transcription factors is regulated to a large extent by post-translational modifications such as phosphorylation and by poly-ubiquitination (Chau et al., 1989; Iwai, 2014), particularly by lys63-linked polyubiquitin chains (Wertz and Dixit, 2010; Iwai, 2014). Proinflammatory signals via TLRs/IL-1R, TNFR, IL-17R, and the T-cell receptor (TCR) lead to Lys63 polyubiquitination of several NF-κB intermediate molecules such as TRAF-6, RIP1, NEMO/IKKγ, and MALT1 (Deng et al., 2000; Tang et al., 2003; Wertz et al., 2004; Chen, 2005; Oeckinghaus et al., 2007), which collectively result in NF-κB activation.

## A20 as a Central Negative Regulator of NF-κB Transcription Factors

The ubiquitin editing protein A20 is encoded by the tumor necrosis factor-α-induced gene 3 (TNFAIP3) and has been shown to negatively regulate NF-κB signaling by multiple mechanisms (Coornaert et al., 2009; Vereecke et al., 2009; Shembade and Harhaj, 2010; Catrysse et al., 2014). Binding of the inflammatory molecules TNFα, IL-1β, LPS, CD40, and IL-17 to their respective cell surface receptors promotes the recruitment of specific adaptor proteins. Some of these adaptor proteins are shared by several signal transduction pathways. The IL-17R for instance, shares many downstream adaptors and transcription factors with the TNFα, IL-1, and TLR pathways. The IL-17R subunits IL-17RA and IL-17RC do not recruit TLR-associated adaptors such as MyD88, but instead associate with the adaptor protein Act1/CIKS. Upon recruitment, Act1 activates pro-inflammatory signaling pathways including TRAF6/NF-κB, MAPK and CCAAT Enhancer Binding Proteins (C/EBP; Gaffen, 2009). Each receptor signaling complex ultimately activates the IκB kinase (IKK) complex. IKK activation, in turn, phosphorylates IκB proteins, which results in their proteasomal degradation, thereby enabling the release and translocation of NFκB into the nucleus and subsequently the activation of NF-κB target genes. A20 has been demonstrated to interact with several molecules within these different signaling pathways including TNF Receptor Associated Factor (TRAF)2, TRAF6, RIP, and IKKγ (Beyaert et al., 2000; Boone et al., 2002; Longo et al., 2003; Garg et al., 2013).

**Figure 1:**
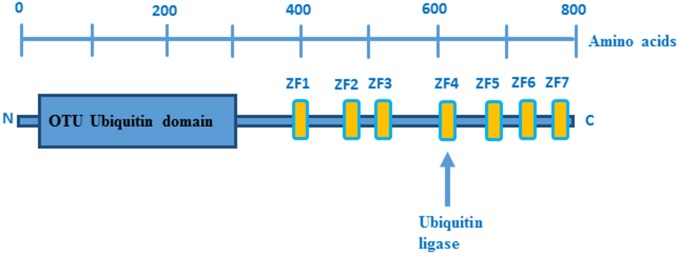
**Domain structure of A20**. OTU ubiquitin protease domain and zinc fingers domains are depicted in blue and yellow boxes, respectively. Picture adopted from Enesa and Evans (2014).

The human TNFAIP3 gene is localized on chromosome 6 and encodes the 790 amino acid protein A20, which is composed of an N-terminal protease domain and seven Cys2-Cys2 zinc finger C-terminal domains (Figure 1). A20 functions as an ubiquitin-editing enzyme (Wertz et al., 2004) and belongs to the ovarian tumor (OTU) proteases family of deubiquitinating (DUB) enzymes. OTU DUB enzymes are a superfamily of cysteine proteases that cleave ubiquitin from branched or linear polyubiquitin chains (Makarova et al., 2000; Evans et al., 2004). In addition, A20 has also an E3 ubiquitin ligase activity that is due to its fourth zinc finger motif in the C-terminal domain (Evans et al., 2004).

While A20 suppressed proximal signaling intermediates of NF-κB activation in cells stimulated with TNFα, IL-1β, LPS, and MDP, it failed to block direct activation of the IKK complex by the viral protein Tax, suggesting that A20 functions upstream of the IKK complex. This hypothesis was confirmed by the observations that A20^−/−^ cells displayed prolonged TNF-induced IKK activity and that these cells were unable to terminate NF-κB transcription (Lee et al., 2000). Although A20 inhibits the TNFR, IL-1R, and NLR pathways, it is not known whether A20 binds directly to any of these receptors. In contrast, A20 was found to inhibit IL-17 signaling by directly binding to the C-terminal domain of IL-17RA (Garg et al., 2013).

**Figure 2:**
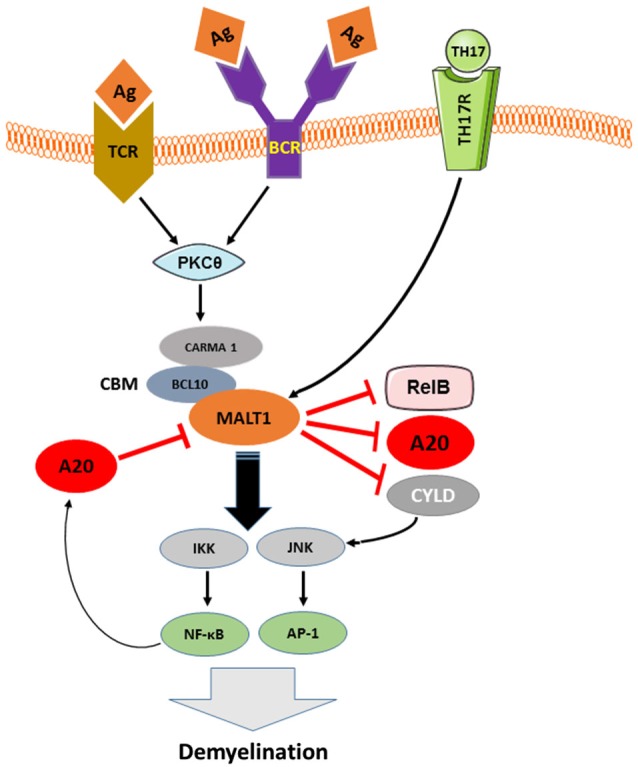
**The pathologic role of TCR, BCR and TH17-induced MALT-1 in demyelination and the inhibition of this signaling pathway by A20**. As depicted in Figure 2, downstream of CBM, IKK complex is activated by MALT-1 that in turn, plays an essential role in the development of EAE-mediated Demyelination. MALT1, in other hand, cleaves the NF-κB inhibitory proteins A20 and CYLD as well as the NF-κB subunit RelB in antigen receptor-stimulated T cells and during EAE, suggesting a contribution of MALT1 proteolytic activity in T cell activation and EAE development. A20, negatively regulates TCR and BCR signaling to NF-κB by cleaving MALT-1 ubiquitin chains and therefore contributes to balance TCR/BCR-induced IKK/NF-KB signaling. Activated A20 might play an important role in protecting neural cells against autoimmune mediated demyelination.

More detailed insights into the mechanisms that underlie NF-κB inactivation by A20 came from the discovery of an OTU domain at the N-terminus of A20 (Evans et al., 2004). Since RIP, TRAF6 and NEMO have been identified as OTU domain substrates (Boone et al., 2004; Wertz et al., 2004; Mauro et al., 2006), and given that these signaling molecules are polyubiquitinated upon recruitment to their respective signaling complexes, it was proposed that A20 attenuates NFκB signaling by deubiquitinating these critical signaling components (Boone et al., 2004; Wertz et al., 2004). Indeed, A20 was shown to attenuate TLR-4/LPS, TNFα, IL-1, IL-17, and NOD2 induced NF-κB signaling by disassembling the lys63-linked polyubiquitin chains from RIP1, RIP2, TRAF6, and NEMO (Boone et al., 2004; Wertz et al., 2004; Hitotsumatsu et al., 2008; Garg et al., 2013). In addition to its deubiquitinating activity, A20 displays ubiquitin ligase activity mediated by its ZF4 domain, which is also involved in NF-κB inhibition. To this end, A20’s E3 ubiquitin ligase activity promotes RIP1 lys48-linked polyubiquitination, which triggers proteasomal degradation of RIP1 and thereby inhibits TNFα-induced NF-κB activation (Wertz et al., 2004). In addition, A20 negatively regulates TCR-induced activation of NF-κB by deubiquitinating Malt1 (Düwel et al., 2009). The adaptor molecule MALT-1 binds to Carma1 and Bcl10 to form the CBM complex, which mediates canonical NF-κB activation (Ruland et al., 2003). In lymphocytes, ubiquitination of MALT-1 by TRAF-6 is a prerequisite for NF-κB activation (Oeckinghaus et al., 2007). The fact that MALT-1 ubiquitination by TRAF6 is essential for TCR- and BCR-induced activation of NF-κB and evidence that MALT-1 plays an important role in the pathogenesis of several autoimmune diseases (e.g., Multiple Sclerosis; Brüstle et al., 2012; Mc Guire et al., 2013) and lymphomas (e.g., Marginal B zone lymphoma and ABC-DLBCL; Ngo et al., 2006; Vega et al., 2011) suggest that MALT-1 inhibition by A20 plays an important role in the prevention of autoimmunity and lymphomas (Figure 2).

## A20 and Apoptosis

Although A20 was initially described as an inhibitor of TNF-induced apoptosis (Opipari et al., 1992), its anti-apoptotic function still remains controversial. This is largely due to the fact that the protective effect of A20 is cell type- and stimulus-specific (Daniel et al., 2004). A20 protects against TNF cytotoxicity in several cell lines including human breast carcinoma MCF7 cells, murine fibrosarcoma WEHI164 cells, murine embryonic fibroblast NIH3T3 cells, mouse osteoblastic MC3T3-E1 cells, and human umbilical vein endothelial cells, but fails to protect HeLa cells and lung epithelial A549 cells (Opipari et al., 1992; Jänicke et al., 1994; Jäättelä et al., 1996; Slowik et al., 1997; Hess et al., 1998; Natoli et al., 1998; De Valck et al., 1999). In addition, A20 protects Jurkat cells from TNFα- but not from Fas-mediated apoptosis (He and Ting, 2002). Moreover, stimulation of B lymphocytes via CD40 induces A20, which in turn, inhibits B cell apoptosis (Sarma et al., 1995). In addition to TNF-induced cell death, A20 has been shown to inhibit cell death induced by various other apoptotic stimuli. A20 protects endothelial cells from Fas/CD95 receptor induced apoptosis, lymphoblastic B cells from apoptosis induced by serum depletion, macrophages from apoptosis induced by oxidized low-density lipoprotein (Sarma et al., 1995; Daniel et al., 2004; Li et al., 2006), H1299 epithelial cells from apoptosis mediated by p53 overexpression (Fries et al., 1996), and the human microvascular endothelial cell line HMEC-1 from apoptosis induced by LPS (Hu et al., 1998). Why A20 protects only selected cell lines from apoptosis remains to be investigated.

The inhibition of TNFα-induced apoptosis by A20 correlates with the inhibition of phospholipase A2, reduced production of reactive oxygen species (ROS), diminished collapse of mitochondrial membrane potential, decreased activation of caspase-3-like proteases and inhibition of JNK (Jäättelä et al., 1996; Wissing et al., 1998; Lademann et al., 2001; Daniel et al., 2004; Won et al., 2010). A20 acts early in the TNFα-induced signaling cascade by blocking both TNFα-induced rapid activation of the c-Jun N-terminal kinase and processing of the receptor-associated caspase-8 (Lademann et al., 2001). Daniel et al investigated the cytoprotective function of A20 in endothelial cells and demonstrated that A20 targets the TNFα–induced apoptotic pathway by inhibiting the proteolytic cleavage of caspase 8 and 2 as well as caspase 3 and 6. Their results also showed that A20 protected endothelial cells from Fas-mediated and natural killer cell-mediated apoptosis through inhibition of Caspase 8 activation (Daniel et al., 2004).

The interaction between A20 and anti-apoptotic proteins such as the TAX-1 binding protein 151 (TXBP151), also suggests a possible survival mechanism. A20 associates with TXBP151 through its C-terminal zinc finger domain, which has been shown to mediate the anti-apoptotic activity of A20 (De Valck et al., 1999). The observation that the over-expression of TXBP151 inhibits TNF-induced apoptosis in some cells as well as the fact that A20 binds TXBP151 may explain at least part of the anti-apoptotic effects of A20. This hypothesis is further strengthened by the observation that transfection with antisense TXBP151 cDNA partially prevents the anti-apoptotic activity of A20 (De Valck et al., 1999).

Another possible mechanism for the anti-apoptotic effects of A20 has been proposed by Won et al. (2010): A20 binds to ASK1, an important MAPKK kinase in the JNK signaling cascade, and mediates ASK1 degradation, leading to suppression of JNK activation and eventually inhibition of apoptosis (Won et al., 2010). It has been well demonstrated that persistent activation of the JNK signaling cascade contributes to TNF-induced apoptosis. Furthermore, A20 may inhibit apoptosis through the suppression of pro-inflammatory cytokines (Nagamachi et al., 2014).

## A20 in Aging and Senescence

Biological aging is related to the accumulation of molecular and cellular damage and accompanying cellular stress response mechanisms. IKK/NF-κB transcription factors are central to these age-related stress-response mechanisms (Solt et al., 2007) as they are activated by inflammatory stimuli, DNA damage and oxidative stress and control anti-oxidative and inflammatory responses, cell survival and apoptosis. Expression of NF-κB transcription factors is strongly associated with age in both mice and humans and genetic blockade of NF-κB in epidermal cells of aged mice reverts their gene expression program to that of young mice (Adler et al., 2007). Further evidence for a role of NF-κB transcription factors in aging and senescence stems from the analysis of progeroid diseases. Progeroid syndromes are a group of genetic disorders characterized by premature development of signs and symptoms typical for normal aging. Mice and humans with defects in certain DNA repair enzymes develop age-related diseases at young age suggesting a role for DNA damage in normal aging (Hasty et al., 2003). This assumption is further supported by similar transcription profiles found in animal models of progeroid syndromes and normally aged mice and humans (Schumacher et al., 2008) as well as the association of NF-κB activity with normal and premature aging (Kriete et al., 2008; Niedernhofer and Robbins, 2008). In addition, signaling via NF-κB transcription factors is altered in age-related diseases including neurodegenerative diseases, diabetes and atherosclerosis (Verma, 2004; Chami et al., 2012; Müller-Rischart et al., 2013; Pranski et al., 2013; Woodling et al., 2014; Zhang et al., 2014). Numerous cell types including blood cells divide for a limited number of divisions and then enter a non-proliferating and apoptosis resistant state, called senescence. The NF-κB transcription factor RelA maintains cellular senescence by facilitating DNA repair mechanisms (Wang et al., 2009) and overexpression of the NF-κB transcription factors RelA and c-rel results in the induction of senescence in cultured cells (Seitz et al., 2000; Bernard et al., 2001, 2004; Wang et al., 2009).

Since A20 is a central inhibitor of these transcription factors, it is conceivable that A20 has a strong impact on normal and premature aging as well as on cellular senescence. Aged mice, for instance, display a reduced immunologic activity of alveolar macrophages and this age-dependent macrophage dysfunction is associated with poor NF-κB activation and elevated expression of A20 (Hinojosa et al., 2014). In addition, mice with B lymphocytes deficient in A20 display elevated serum levels of IL-6, enhanced proliferation of myeloid cells and T lymphocytes and develop autoimmune pathology (e.g., splenomegaly, plasma cell hyperplasia and presence of class-switched autoantibodies) with advanced age (Chu et al., 2011). The role of A20 in aging and senescence is only beginning to be explored and warrants further investigation.

## A20 in Autophagy

The maintenance of cellular homeostasis necessitates mechanisms to degrade and remove cellular components that are no longer required or dysfunctional. Autophagy is the central catabolic mechanism of the cell and involves targeting of unwanted components to autophagosomes, specialized intracellular compartments, which fuse with lysosomes for further degradation. Recently, A20 has been shown to regulate autophagy triggered by the LPS-receptor TLR4. TLR4 signaling leads to association of the adaptor protein TRAF-6, an E3 ubiquitin ligase and scaffold protein, with Beclin 1, a key component of a class III phosphatidylinositol 3-kinase complex (PI3KC3). As a consequence of association with TRAF-6, Beclin-1 is ubiquitinated (K63), facilitating the oligomerization of Beclin 1 and the activation of PI3KC3. This triggers the formation of autophagosomes and induces autophagy in macrophages (Shi and Kehrl, 2010). Consequently, A20 restricts the TLR4-induced autophagy through the reduction of lys63-linked ubiquitination of Beclin 1 (Shi and Kehrl, 2010).

A20 has also been shown to inhibit the TNFα-mediated JNK1 activation, an important autophagy inducing pathway (Bubici et al., 2006). Activation of the JNK1 pathway stimulates the dissociation of the Bcl-2/Beclin 1 complex, leading to enhanced autophagy (Wei et al., 2008). In addition, A20 down-regulates the activity of the autophagy receptor NDP52 (Inomata et al., 2012), which plays an important role in the selective elimination of pathogenic bacteria. During the elimination of microbes, autophagy appears to protect cells from excess innate immune signaling that often generates a cytotoxic level of ROS (Thurston et al., 2009; Cemma et al., 2011).

It has also been shown that NDP52 exerts a negative regulatory effect on TLR-induced pro-inflammatory responses through degradation of activated TLR adaptor molecules and of TRAF6 (Inomata et al., 2012). In this case, NDP52 mediates aggregation of the complexes MyD88–TRAF6 and TRIF–TRAF6. This process is important for selective degradation of unnecessary signaling molecules in lysosomes via an autophagic mechanism. The NDP52-mediated autophagy ultimately leads to negative regulation of TLR signaling, suggesting that in this case activated autophagy could be an important mechanism to maintain homeostasis in the innate immune system. However, this effect of NDP52 could be inactivated by A20, suggesting that A20 acts as a negative regulator of the negative regulatory effect of NDP52 (Inomata et al., 2012).

## A20 in Lymphomas

Lymphomas are the fifth most common human malignancy and the vast majority of lymphomas originate from the B cell lineage. Mutations in NF-κB and JAK/STAT signaling proteins cause defects in B cell survival and proliferation, and contribute to lymphomagenesis (Malynn and Ma, 2009). Since these signaling cascades contain some ubiquitin-dependent signaling molecules (e.g., TRAF-6), it is possible that A20 may also regulate B cell homeostasis and activation, although the majority of studies investigating the role of A20 in immune responses have focused on the innate immune system.

Considering the association of chronic NF-κB activation with tumorigenesis, it is not surprising that A20 is commonly inactivated in tumor cells. A20 inactivation up-regulates NF-κB-induced pro-survival genes, leading to uncontrolled proliferation of tumor cells and enhancement of their resistance to apoptosis (Hymowitz and Wertz, 2010). However, reintroduction of A20 into A20-deficient tumor cell lines, leads to the induction of cellular apoptosis and the suppression of NF-κB activity (Honma et al., 2009). It has been shown that A20 is the most commonly affected gene in diffuse large B cell lymphomas and lymphoplasmacytic lymphomas (Compagno et al., 2009; Braggio et al., 2012). For instance, A20 is cleaved and inactivated by MALT1 following TCR stimulation. MALT1 is a functional cysteine protease that is constitutively active in several lymphomas such as mucosa-associated lymphoid tissue lymphoma (MALT lymphoma; Coornaert et al., 2008). A20-deficient cells stably generated tumors in immunodeficient mice, whereas the tumorigenicity was effectively suppressed by re-expression of A20 (Kato et al., 2009). Consistent with this, expression of A20 has been shown to cause cell death in A20 deficient lymphoma cell lines but not in A20 competent lymphomas (Schmitz et al., 2009).

A20 can be inactivated by promoter methylation, somatic mutations and genomic deletions in various numbers of lymphomas such as marginal zone lymphomas, MALT lymphoma, primary mediastinal B cell lymphomas (PMBLs) and Hodgkin’s lymphoma (Honma et al., 2008; Compagno et al., 2009; Kato et al., 2009; Novak et al., 2009; Chanudet et al., 2010) and the loss of A20 protein due to bi-allelic mutations of TNFAIP3 occurs in some Hodgkin lymphomas and primary mediastinal lymphomas (Schmitz et al., 2009).

Recently, the role of A20 in tumor suppression was demonstrated in the primary cutaneous T cell lymphoma Sézary syndrome (SS; Braun et al., 2011). Mono- and biallelic deletions of A20 were found in 46% (6/13) of SS-patients and reconstitution of A20 in an A20 deficient SS cell line suppressed cell proliferation (Braun et al., 2011). Collectively, these findings demonstrate that A20 may act as a tumor suppressor in human lymphomas.

## The Role of A20 in Autoimmune Diseases Outside the CNS

NFκB transcription factors play a key role in the regulation of self-directed and protective immune responses and perturbation or excessive activation of NFκB signaling is associated to a number of autoimmune and chronic inflammatory diseases (see the review by Sun et al., 2013). A20 is one of the most potent inhibitors of NFκB signaling and dysfunction of A20 results in excessive inflammation and autoimmunity. Accordingly, mice genetically deficient in A20 develop a lethal autoimmune disease, characterized by increased responsiveness to TNFα- and TLR-mediated signals and spontaneous inflammation within multiple organs including liver, kidneys, intestines, joints and bone marrow (Lee et al., 2000; Boone et al., 2004).

In humans, single nucleotide polymorphisms (SNPs) of the TNFAIP3 gene have been associated with a range of autoimmune diseases including systemic lupus erythematosus (SLE), rheumatoid arthritis (RA), inflammatory bowel disease, psoriasis and type one diabetes (Musone et al., 2011; Ma and Malynn, 2012). SNPs located within the coding region of genes may either be silent or affect protein sequence, structure and function. More often, SNPs lie within the noncoding regions of the gene and may affect gene transcription, splicing or RNA stability. The wide tissue distribution of A20 is likely to account for the broad spectrum of clinical phenotypes and the contribution of A20 expressed in individual cell types to the development of autoimmune diseases has thus been addressed in a range of conditionally knock out animals.

Genome wide association studies (GWAS) identified several SNPs within the TNFAIP3 gene and the A20 interacting protein ABIN1 (Gateva et al., 2009) in SLE (Musone et al., 2008). Because of the broad tissue distribution of A20 and the lethality of A20 deficient mice, a range of conditionally knockout animals have been generated to study tissue-specific deletion of A20. Mice lacking A20 specifically in CD11c+ dendritic cells show phenotypical similarities to human SLE including glomerulonephritis, presence of antibodies against dsDNA, ribonucleoprotein and cardiolipin, thrombocytopenia and autoimmune lymphoproliferative syndrome (ALPS) including enhanced spontaneous lymphocyte activation, accumulation of double negative lymphocytes and myeloproliferative syndrome (Kool et al., 2011). These data link A20 expression in dendritic cells to SLE and this issue will have to be further addressed.

In addition, several SNPs in the A20 genomic locus have been associated with RA (Bowes et al., 2010; Shimane et al., 2010), but their relevance in disease pathogenesis has not yet been determined. Mice deficient for A20 in myeloid cells display inflammation within the joints, cartilage destruction and anti-collagen antibodies but no obvious inflammation at other sites including skin, liver, intestines and pulmonary system. This phenotype is similar to human RA (Matmati et al., 2011). A20 deficient macrophages showed an increased degradation of the NF-κB inhibitory molecule IκBα and produced larger quantities of TNFα in response to TLR4 activation. Myeloid cells are the main source of TNFα and TNFα plays a crucial role in the pathogenesis of RA as demonstrated by the effectiveness of TNFα blocking agents. Collectively, these data indicate that A20 in cells of the myeloid lineage may be involved in RA pathogenesis. In line with this, synovial fibroblasts from RA patients display prolonged inflammatory cytokine production and reduced expression of the inhibitory protein ABIN-3 in response to stimulation with TNFα (Lee et al., 2013).

The common human inflammatory skin disorder psoriasis is believed to result from an imbalance of the tightly regulated homeostasis between keratinocytes, mesenchymal cells and immune cells within the skin (Nestle et al., 2009). TNFα mediated NF-κB activation plays a central role in the pathogenesis of psoriasis (Kumari et al., 2013) as illustrated by the responsiveness to anti-TNFα therapy (Lowes et al., 2007). SNPs in the TNFAIP3 gene, but also in other immune-regulatory genes including HLA-C, TNIP1/ABIN-1 and IL-23A (Nair et al., 2009; Strange et al., 2010; Johnson-Huang et al., 2012) have been associated to psoriasis and polymorphisms in the TNFAIP3 gene correlate to responsiveness to TNFα blockers in psoriasis patients (Tejasvi et al., 2012). In animal models, inhibition of NF-κB activity by deletion of IκB kinase 2 in keratinocytes induces inflammatory skin lesions (Pasparakis et al., 2002) while ablation of A20 in skin keratinocytes results in keratinocyte hyperproliferation but not inflammation (Lippens et al., 2011). In contrast, genetic ablation of ABIN-1 in CD11c+ dendritic cells increases experimental psoriasis in mice (Callahan et al., 2013). These data suggest that A20 or A20 interacting proteins could be impaired in dendritic cells of psoriatic patients.

## A20 in Brain Autoimmune Disease

Multiple sclerosis (MS) is the most prevalent of all autoimmune diseases of the central nervous system (CNS) that include neuromyelitis optica, limbic encephalitis and rasmussens encephalitis (Waubant and Cross, 2014). MS is characterized by infiltration of T lymphocytes, B lymphocytes and macrophages into the CNS and immune-mediated destruction of CNS myelin and neurons. In about 70% of patients, MS initially follows a relapsing remitting course, which is accompanied by spontaneous increase and decrease of inflammatory activity within the CNS. The cause of MS and the factors that regulate inflammatory activity are only poorly understood. Low vitamin D serum concentrations, tobacco smoking, Epstein Barr Virus and high sodium intake have been identified as risk factors for MS and more than 100 gene variants have been associated with the disease, but each showing only a weak correlation (Beecham et al., 2013). Among these, SNPs within the TNFAIP3 gene have been associated to MS in a meta-analysis of genome-wide association scans that included 2624 subjects with MS and 7220 control subjects (De Jager et al., 2009).

NF-κB transcription factor expression was found to be increased in macrophages of MS patients and down regulated after treatment with interferon beta, a drug that suppresses MS disease activity (Christophi et al., 2009). Consistent with this, TLR induced cytokine production was increased in MS patient’s macrophages (Christophi et al., 2009). These data would fit to the hypothesis, that MS is related to diminished function of A20 in macrophages. To date, this issue has only been addressed in a single study that found reduced levels of A20 mRNA in monocytes and to a lesser extent in CD4+ helper T cells in patients with MS (Navone et al., 2014). Further research could clarify whether A20 protein is altered in macrophages and possibly other types of antigen presenting cells in MS patients and whether A20 expression correlates to different stages or activity of the disease or responsiveness to specific immunomodulatory treatment. In this context it is important to note that TNFα directed therapies do not ameliorate MS disease activity. While anti-TNFα therapy is exquisitely effective in RA, psoriasis and inflammatory bowel disease, it can worsen MS disease activity (The Lenercept Multiple Sclerosis Study Group and The University of British Columbia MS/MRI Analysis Group, 1999) or induce MS in individuals treated for systemic autoimmune conditions (Hare et al., 2014). A further clarification of the role of A20 and A20 related molecules in these conditions could thus provide important insights into the specific underlying disease mechanisms and provide new concepts for individualized therapy.

In experimental autoimmune encephalomyelitis (EAE), the animal model of MS, NF-κB transcription factors critically determine disease activity. Inhibition of NF-κB by blocking of IKK protects mice from EAE (Dasgupta et al., 2004). Due to the broad tissue distribution of NF-κB, it is obviously hard to experimentally attribute this effect to specific cell types, i.e., immune cells vs. CNS resident cells. Mice deficient for p50-NF-κB display markedly reduced EAE disease severity and reduced peripheral T cell activation and differentiation, while the numbers of CNS infiltrating immune cells (CD3+ T cells, B220+ B lymphocytes and CD11b+ macrophages) in mice with severe EAE is significantly increased in NF-κB1 deficient animals as compared to wild-type littermates (Hilliard et al., 1999).

In line with this, mice deficient in the NF-κB regulator MALT1 do not develop EAE, despite strong lymphocytic infiltration into the CNS while adoptively transferred wild type T helper cells efficiently induce EAE in MALT1^−/−^ hosts. MALT1 deficient Th17 cells show reduced expression of the effector cytokines IL-17 and GM-CSF (Brüstle et al., 2012). Collectively, these data point towards a role for NF-κB in immune cells as opposed to CNS cells in the regulation of CNS-directed immunity. In spite of these observations, NF-κB also largely regulates activity of neurons and glia cells during CNS inflammation. Inactivation of NF-κB specifically in astrocytes leads to improved clinical outcome of EAE and reduced expression of inflammatory cytokines within the CNS in mice (Brambilla et al., 2014). In line with this, mice lacking A20 in astrocytes display increased EAE disease severity (Wang et al., 2013) and A20 deficient astrocytes showed increased activity of NF-κB and produced larger amounts of chemokines in response to stimulation with the inflammatory cytokines TNFα, IL17 and GM-CSF. These data consistently provide evidence for a role of NF-κB in astrocytes in the regulation of CNS autoimmunity and astrocyte-mediated neurotoxicity.

## A20 and Neurodegeneration

**Figure 3:**
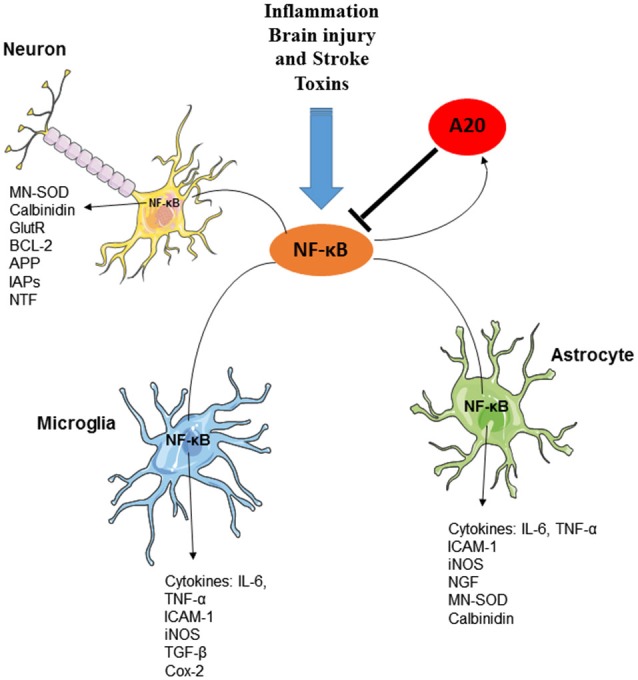
**The essential role of A20 in the regulation of NF-κB in neurons, astrocytes and microglia**. Several factors including inflammation, brain injury and stroke and toxins can induce the production and activation of NF-κB transcription factor in various cells of CNS. Activation of NF-κB, results in the induction of several inflammatory mediators as well as the activation of Ubiquitin-editing enzyme A20. A20, in turn, negatively regulates NF-κB signaling and preserves CNS-specific homeostasis.

Functional NFκB complexes are present in neurons, astrocytes, microglia, and oligodendrocytes of the CNS (O’Mahony et al., 2006; Camandola and Mattson, 2007; Figure 3). Interestingly, similarly to B cells, neurons exhibit high constitutive NFκB activity; NFκB is present in synapses, where it is readily available to respond to synaptic stimulation. In fact, it is to date the only transcription factor that has been shown to be activated locally in synapses (Meffert et al., 2003; Marcora and Kennedy, 2010). NFκB is, contrary to many other synaptically relevant transcription factors, responsive to low-frequency stimulation coupled with long-term depression (LTD) and it is further activated in the context of long-term potentiation (LTP; Meffert et al., 2003; Oikawa et al., 2012). Various neurotransmitters, including glutamate, Ca^2+^ influx, ROS and cytokines, such as TNF and IL-1, and β-amyloid precursor protein stimulate receptors mediating NFκB activation in neurons (Albensi and Mattson, 2000; Kaltschmidt et al., 2005). Consequently, an important role for NFκB has been demonstrated in the regulation of neurogenesis, neuritogenesis, synaptogenesis, and of learning and memory (O’Mahony et al., 2006; Zhang and Hu, 2012; Methot et al., 2013; Mihalas et al., 2013). In contrast, in astrocytes or in microglia, NFκB signaling does not appear to be constitutively active, nor is it responsive to activation by calcium, but is instead readily induced by proinflammatory molecules, accompanied by the production of a multitude of cytokines and chemokines, eicosanoids and reactive nitrogen and oxygen species by glial cells.

Not surprisingly, NFκB has been implicated in the pathogenesis of Parkinson’s disease (PD), Huntingdon’s disease (HD), Alzheimer’s disease (AD), amyotrophic lateral sclerosis (ALS), MS and stroke (Kaltschmidt et al., 1999; Dasgupta et al., 2004; van Loo et al., 2006; Ghosh et al., 2007; Sarnico et al., 2009; Marcora and Kennedy, 2010; Baiguera et al., 2012; Hsiao et al., 2013). Much of the role of NF-κB in neurodegenerative diseases stems from its part as a regulator of inflammatory actions either in immune cells or in macroglia. Nonetheless, the role of NF-κB in the regulation of synaptic efficacy should not be ignored either, when considering the ultimate etiology of neurodegenerative diseases (Dasgupta et al., 2004; Ghosh et al., 2007; Marcora and Kennedy, 2010). Dissociated cultures of hippocampal neurons from mice exhibiting delayed synthesis of IκBα are hyperexcitable, whereas persistent IκB kinase complex (IKK)/NF-κB signaling in forebrain neurons does not induce overall neuroinflammation, but elicits a selective inflammatory response in the dentate gyrus of the hippocampus, accompanied by decreased neuronal survival and apoptosis, independent of neurodegeneration (Maqbool et al., 2013). In line with this, enhanced IKK-NFκB signaling upon inflammation in HD astrocytes plays a detrimental role in neuronal survival in the Htt mouse model and in HD patients (Hsiao et al., 2013). On the other hand, inhibition of IKK complex in microglia and astrocytes within the substantia nigra pars compacta, but not in neurons, where NF-κB activity is also upregulated, improves motor functions in the MPTP mouse model of PD (Ghosh et al., 2007). Furthermore, reduced nuclear localization of the Rel-A (p65) subunit of the NFκB dimer confers protection in the study mentioned above, while a deficiency in the c-Rel subunit of NFκB renders mice to develop parkinsonism with aging, characterized by microglia, but not astrocyte, activation (Baiguera et al., 2012). Finally, in the middle cerebral artery occlusion (MCAO) model of stroke the outcome of the insult has been shown to depend on the NFκB dimer subunit composition in the affected tissue: c-Rel is in general anti-apoptotic and target genes of c-Rel include several antioxidant and anti-apoptotic genes, whereas RelA-containing NF-kB dimers facilitate apoptosis (Sarnico et al., 2009). Taken together, it appears that the majority of the detrimental NFκB activation in CNS disorders takes place in micro- and macroglia cells, not in neurons. Moreover, some of the discrepancies in the literature concerning the role of NFκB in neurodegenerative diseases may result from the formation of NFκB dimers of different subunit composition in each particular model system applied, followed by activation of a characteristic set of target genes. Notably, the NFκB target genes activated during basal synaptic transmission in neurons have not yet been identified.

How the activity of the NFκB dimer is regulated in the CNS remains poorly understood. Different cell types within the CNS may putatively utilize cell type and cell sub-type specific regulators reflecting the unique functions of NFκB in glial cells as compared to neurons. Recent reports provide evidence that A20 is crucial for NF-κB pathway regulation in CNS disorders. Astrocyte restricted deletion of A20, but not a similar deletion in neurons, aggravated the outcome of EAE leading to chemokine overproduction, increased numbers of encephalitogenic CD4+ T lymphocytes and cytokine production, enhanced recruitment of inflammatory leukocytes and demyelination in the spinal cord (Wang et al., 2013). Additionally, SNP of A20 have been linked to susceptibility to MS (De Jager et al., 2009). In clinical studies decreased A20 mRNA expression was detected in MS patients compared to control individuals. Low A20 amounts are indicative for a more severe course of the disease (Gilli et al., 2011). Furthermore, increased cerebrospinal fluid (CSF) levels of the chemokine CXCL13, a prognostic MS marker, have been reported for MS patients with A20 SNPs (Lindén et al., 2013). A20 has also been implicated in the regulation of NF-κB activation in a microglia cell line, though *in vivo* evidence for the role of A20 in the regulation of microglia NF-κB activity is still missing (Dalal et al., 2012). Finally, studies exploring the function of A20 in NF-κB regulation upon MCAO have different conclusions, one stating reduced infarct volume and improvement of neurological deficits in A20 gene deficient rats, though whether these effects were due to reduced NF-κB activity was not examined (Yu et al., 2006). However, another work employing knockout mice for A20, either in the CNS or specifically in neurons, did not detect differences in infarct volume when assessed 24 h after MCAO, although MCAO upregulated A20 expression in wild type mice (Mc Guire et al., 2013).

Consequently, some initial evidence exists to support the proposal that A20 would regulate NF-κB activation in the CNS. To investigate this, Pranski and colleagues examined the expression of “NF-κB editing complex” components, A20, RING finger protein 11 (RNF11), Itch (an E3 ligase) and Tax binding protein (TAX1BP1), in the normal human brain by quantitative PCR (RT-PCR) and immunohistochemistry (IHC; Pranski et al., 2013). Both A20 and RNF11 were detected by RT-PCR in the samples of the frontal cortex, striatum, hippocampus, pons and medulla. By IHC predominantly neuronal but also glial expression was reported for RNF11, Itch and TAX1BP1 in the brain structures mentioned above. Unfortunately, A20 protein itself was not detectable with the antibodies used and the putative cell type specific expression of A20 in the healthy CNS remains an unresolved issue. Previously, RNF11 has been demonstrated to localize to Lewy bodies in Parkinson disease brain (Anderson et al., 2007). Using murine primary neurons depleted for RNF11, the same lab also showed that RNF11 depletion leads to nuclear localization of RelA (p65) (Pranski et al., 2013). Nonetheless, RNF11-mediated inhibition of NF-κB was found to exacerbate 6-OHDA toxicity, a PD disease model, in *in vivo* and *in vitro* experiments, whereas reduced expression of RNF11 and consequently increased NF-κB activation protected against neurodegeneration (Pranski et al., 2013).

Taken together, A20 is emerging as an important regulator of NF-κB activation in astrocytes and possibly in neurons upon insult. Whether it may contribute to the regulation of constitutive NF-κB activity in neurons or in neurodegenerative processes is currently an open question. Both in microglia and in astrocytes, NF-κB activation is usually transient, whereas neurons exhibit sustained NF-κB activity. Persistent activation is usually associated with inflammation. This may be reflected in the manner NF-κB activity is regulated in neurons vs. glial cells and in the molecules involved. Two other A20 related deubiquitinating cysteine proteases Cezanne 1 (Cellular zinc finger anti- NF-κB Otud7b/Ovarian tumor domain) and Cezanne 2 (Otud7a) were identified on the basis of their overall sequence similarity to A20 and due to their potential to regulate NF-κB signaling and were hence termed to belong to “A20 family of proteins” (Evans et al., 2001; Enesa et al., 2008). The molecules share the N-terminal OTU domain that mediates deubiquitination and regulation of NF-κB signaling. Cezanne also bares a single conserved zinc finger motif that is similar to the A20 zinc finger motifs conferring E3 ubiquitin ligase activity to A20 (Evans et al., 2001; Ma and Malynn, 2012). Expression of all Cezanne molecules, like that of A20, is induced by NF-κB creating thereby a negative feedback loop for NF-κB signaling (Luong et al., 2013).

Yet, although A20 and Cezanne possess overlapping structural and biochemical properties, they exert unique functions by targeting distinct forms of poly-ubiquitin (Bremm et al., 2010). This, together with the fact that their cell and tissue specific expression profiles differ, imply that A20 and Cezanne molecules might have to some extent overlapping, yet simultaneously unique functions as regulators of NF-κB signaling. Cezanne 1 is ubiquitously expressed in various cell types and tissues, though the brain seems to exhibit the highest Cezanne 1 mRNA levels (Hu et al., 2013; Luong et al., 2013). Cezanne 2 expression is instead restricted to the central nervous system (CNS), where it is particularly abundant in the cerebral cortex and in the cerebellum (Hu et al., 2013). On the other hand, A20 is specific to immune cells, yet inducible in pathological conditions in various other cell types including macroglia (Wang et al., 2013).

Using Cezanne 1 knockout mice, a recent study revealed Cezanne 1 as a specific regulator of non-canonical NF-κB signaling (Hu et al., 2013). Cezanne 1 deficiency in mice does not result in a significant effect on canonical NF-κB activation, but causes hyper-activation of the non-canonical NF-κB pathway. It was also noted that Cezanne 1 does not affect the basal activation of non-canonical NF-κB signaling, but controls only signal-induced NF-κB activity. These observations are of interest as A20 is specific for the canonical NF-κB pathway and no other negative regulator of the non-canonical NF-κB signaling has been identified this far. Nonetheless, some other works have identified Cezanne 1, whose expression is up-regulated by hypoxia, as a negative regulator of the canonical NF-κB signaling, among others in an ischemia-reperfusion model (Enesa et al., 2008; Luong et al., 2013). These initial findings, together with the fact that Cezanne 1 regulates nuclear localization of DJ-1/Parkin 7, mutated forms of which are associated with the early-onset form of PD (McNally et al., 2011; Saito et al., 2014), may suggest potential roles for Cezanne proteins in the regulation of NF-κB activity in inflammatory responses to ischemia, and in neurodegenerative diseases.

### The Role of A20 in Brain Tumors

Evidence from animal models and human studies support the concept that chronic inflammation promotes sustained NF-κB activation, which in turn facilitates tumorigenesis in a large variety of tissues (Conti et al., 2005; Mattson and Meffert, 2006). The role of A20 in the development of tumors of the CNS is less clear and controversial. A20 was found to be largely overexpressed in human glioblastoma stem cells (GSCs) and glioma tissue samples (Guo et al., 2009; Hjelmeland et al., 2010) and siRNA-mediated downregulation of A20 in U87 glioblastoma cells as well as in a mouse Xenograft tumor model led to significantly reduced tumor growth and tumor size (Guo et al., 2009). In line with this, inhibition of A20 expression in glioma stem cells decreased their growth and their tumorigenic potential (Hjelmeland et al., 2010). In addition, down-regulation of A20 has been correlated to the development of multiple drug resistance (MDR) in glioblastoma cells (Bredel et al., 2006) MDR was proposed to be mediated by a RIP-dependent signaling cascade that leads to NF-κB-induced resistance formation and glioblastoma cell survival. In contrast to these results, expression of A20 was negatively correlated to clinical outcome in a cohort of glioblastoma patients (Bredel et al., 2006).

A possible mechanism for A20’s tumor enhancing function in glioblastoma and GSCs might be the A20-induced apoptotic resistance. Since A20 is a potent anti-apoptotic molecule, it sustains the resistance of gliomas to apoptosis and promotes their proliferation. In glioma cells, A20 displays strong anti-apoptotic activity, even though it inhibits the anti-apoptotic activity of NF-κB (Guo et al., 2009). Taken together, A20 contributes to cell survival in glioma cells and may be an attractive target for glioma therapy. Its function may well vary in different tumors of different cellular origin.

### A20 in Cerebral Ischemia and Post-Ischemic Apoptosis

Brain infarction is accompanied by ischemic tissue damage as well as infiltration of inflammatory cells and immune-mediated neuronal damage (Iadecola and Alexander, 2001). The central role of NF-κB in ischemic tissue damage and associated inflammatory mechanisms is well established and indicates a potential role for A20 in the control of ischemia-induced brain damage. Although A20 is up-regulated in the brain of mice after permanent MCAO, deletion of A20 in either neurons or CNS cells does not alter ischemia-induced tissue damage (Mc Guire et al., 2013). However, over-expression of A20 in primary rat hippocampal neurons and SH-SY5Y cells has been shown to result in the reduction of infarct volume and improvement of neurological deficits following experimentally induced cerebral ischemia (Yu et al., 2006). Furthermore, inhibition of TNFα-induced apoptosis in primary rat hippocampal neurons has also been demonstrated, suggesting a possible neuroprotective function of A20 in ischemic damage.

### Final Conclusion

A20 has been firmly established as a central negative regulator of NF-κB transcription factors but its impact on cellular and organismic functions is only beginning to be assessed. A20 inhibits apoptosis in several cell types, enhances cell survival and has implications for cellular senescence and aging. It appears to act as a tumor suppressor in lymphomas and deletion of A20 within the immune system results in enhanced inflammation and spontaneous autoimmunity. Impaired A20 function in antigen presenting cells has been associated with human autoimmune diseases including psoriasis, RA and MS, but the underlying cellular and molecular mechanisms remain to be addressed. Within the CNS, A20 and the A20 related deubiquitinating proteases Cezanne 1 and Cezanne 2 probably have partially overlapping functions in the regulation of NF-κB in astrocytes and possibly in neurons. Initial results suggest yet to be determined roles of A20 and Cezanne 1 in cerebral ischemia, CNS inflammation and neurodegenerative diseases. The role of A20 in CNS autoimmune disease is largely unknown. However, indirect evidence indicates that A20 function may be impaired in macrophages in MS, which is in line with the observation, that NF-κB in astrocytes and microglia is particularly responsive to inflammatory stimuli. NF-κB has been implicated in the pathogenesis of a variety of neurodegenerative diseases and A20, a key regulator of NF-kB activity, as well as additional A20-related proteases are likely to be involved in Alzheimer’s disease, Parkinson’s disease and stroke.

## Conflict of Interest Statement

The authors declare that the research was conducted in the absence of any commercial or financial relationships that could be construed as a potential conflict of interest.
